# Bile-stained amniotic fluid: a case report

**DOI:** 10.1186/s13256-017-1419-8

**Published:** 2017-09-06

**Authors:** Surasak Puvabanditsin, Charlotte Wang Chen, Suja Vinod, Meghan S. Mehta, Omer Choudry, Lauren Walzer

**Affiliations:** 1 Department of Pediatrics, Rutgers Robert Wood Johnson Medical School, One Robert Wood Johnson Place, New Brunswick, NJ 08903 USA; 2 College of Arts and Sciences, Boston University, Boston, USA

**Keywords:** Intestinal obstruction, Amniotic fluid, Bile-stained amniotic fluid, Neonate

## Abstract

**Background:**

Green-stained amniotic fluid does not always indicate that meconium was passed *in utero*.

**Case presentation:**

We report the case of a 2280-g Hispanic preterm female born at 32 weeks of gestation with congenital jejunal atresia. The amniotic fluid was greenish stained; the initial impression was meconium-stained amniotic fluid. Postnatal findings revealed no meconium in her rectum. The content of her first stool appeared sticky and white.

**Conclusion:**

In the absence of meconium in the rectum, the pediatrician should consider the possibility that the greenish amniotic fluid is not meconium stained, but rather stained with bile due to the fetus vomiting *in utero* secondary to intestinal obstruction.

## Background

Amniotic fluid (AF) can be stained green by bile pigments if the fetus has hemolytic disease, passes meconium, or vomits bile *in utero*. In 1972, the first case reported of bilious vomiting *in utero* was in a neonate with an atretic jejunum [1]. If there is green-stained AF and the baby lacks meconium in the rectum, clinicians should be aware of the possibility of intestinal obstruction. There may be a delay in diagnosing intestinal obstruction in a newborn because of the assumption that the green AF was due to meconium passed *in utero*. Our case highlights the fact that green-stained AF could be due to bile secondary to *in utero* bilious vomiting, and not necessarily due to meconium.

## Case presentation

**Fig. 1:**
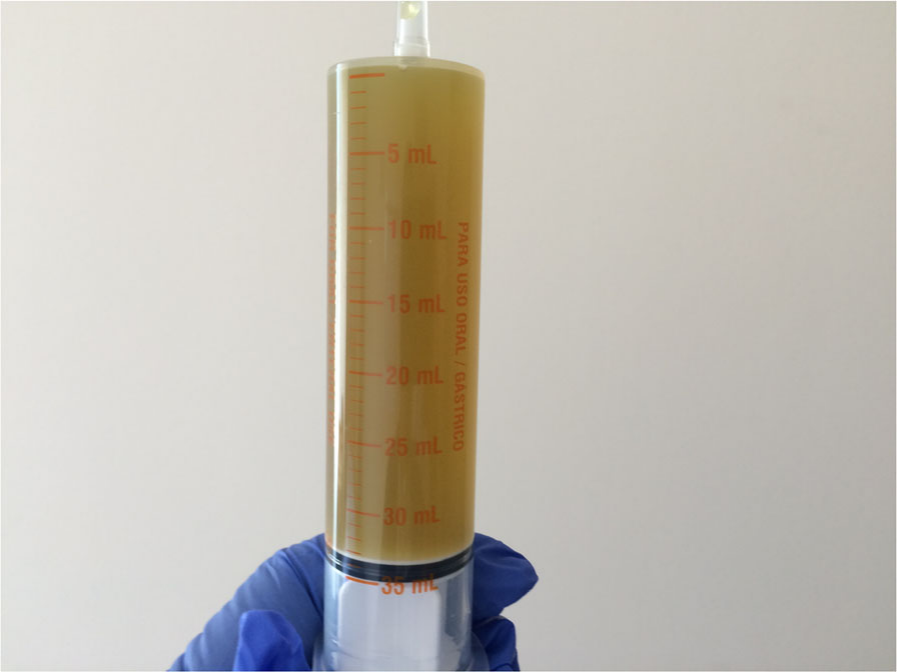
Greenish-stained amniotic fluid

**Fig. 2:**
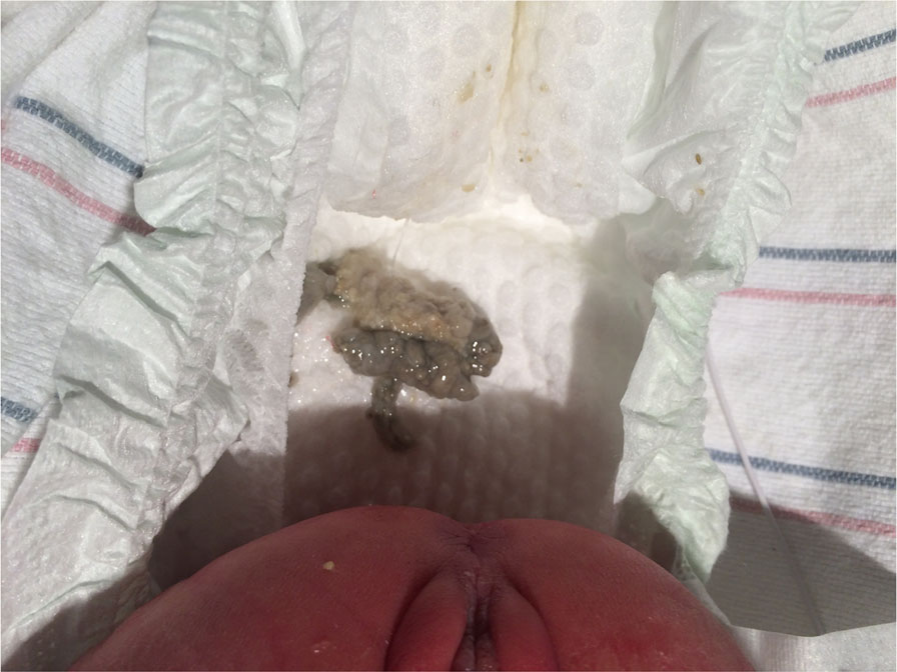
White sticky meconium

A 2280-g Hispanic preterm female was born at 32 weeks of gestation to a 29-year-old gravida (G) 2 para (P) 1 woman by spontaneous vaginal delivery. At delivery, the AF was noted to be “meconium stained.” A total of 35 mL of greenish AF was aspirated from the baby’s stomach (Fig. 1). She had Apgar scores of 9 and 9 at 1 and 5 minutes respectively. The pregnancy was significant for prenatal diagnosis of small bowel obstruction at 31 weeks of gestation. A physical examination revealed a weight of 2280 g (85th centile), length of 48 cm (95th centile), and head circumference of 29 cm (40th centile). She did not have any respiratory distress and there was no abdominal distension. An additional 35 mL of greenish fluid was aspirated from her stomach in the neonatal intensive care unit (NICU). She had her first bowel movement at 48 hours of age; the stool appeared sticky and white (Fig. 2). An upper gastrointestinal (UGI) series and a Gastrografin (sodium diatrizoate and meglumine diatrizoate) enema showed jejunal atresia and microcolon. She underwent exploratory laparotomy on the 3rd day of life. An intervening segment was noted between the two proximal jejunal atresias measuring approximately 10 cm. The jejunal atretic segment was resected and a tapering enteroplasty of that jejunal segment was performed. Her postoperative course was uneventful; she was discharged home at 31 days of age.

## Discussion

Approximately 10% of pregnancies have meconium-stained AF at delivery [2, 3]; however, one quarter of these cases have no evidence of hypoxia. Peristaltic activity has been reported to occur in the fetal bowel as early as 8 weeks of gestation [4], and fetal defecation is a routine physiological event in early and mid-pregnancy [5]. The fetus routinely defecates *in utero* until 16 weeks gestation and finally ceases to defecate by 18 to 20 weeks [6]. Babies born with an anorectal malformation usually have a large dilated rectosigmoid portion of the distal bowel full of meconium, which suggests that there is prevention of fetal defecation *in utero* [5].

Shrand [1] first reported a case of *in utero* bilious vomiting in a neonate with an atretic jejunum. A year later, Daw (1973) described a mother with “golden liquor amnii” when her fore waters ruptured at 36 weeks of gestation [7]. Her live-born baby had an open 6 cm-diameter enterocele that contained the stomach, small intestine, and almost half of the large bowel [7]. Since then, there have been several reports of bile-stained AF (BSAF) in babies with intestinal obstruction (Table 1). Williams *et al*. [8] described a baby with congenital jejunal and ileal atresia, and meconium-stained AF due to *in utero* bilious regurgitation or vomiting. In 1988, two reports described green AF in five babies due to *in utero* bilious vomiting secondary to intestinal obstruction [9, 10]. Akindele (1994) reported a case of a preterm baby born to a teenage mother with fresh “meconium-stained” AF. The baby had a copious amount of green effluent in the pharynx and stomach, and was found to have ileal atresia. The “meconium-stained” AF was due to *in utero* bilious vomiting, secondary to the intestinal obstruction [11].

**Table 1:** Clinical details of 13 neonates with bile-stained amniotic fluid

Diagnosis	Gestational age (weeks)	Birth weight (g)	Authors
Jejunal atresia	Preterm	N/A	[14] Goedvolk and Yap, 2004
Imperforated anus	Term	4220	[15] Vijayakumar and Koh, 2001
High intestinal obstruction	N/A	N/A	[16] Swarte *et al*., 1997
High intestinal obstruction	N/A	N/A	
Ileal atresia	34	2100	[11] Akindele, 1994
Congenital intestinal obstruction	N/A	N/A	[10] Archer, 1988
Congenital intestinal obstruction	N/A	N/A	
Jejunal atresia	Term	N/A	[9] Griffith and Burge, 1988
Meconium peritonitis	32	N/A	
Posterior urethral valve/Microcolon	35	N/A	
Jejunal/Ileal atresia	36	2300	[8] Williams *et al*., 1978
Enterocele	36	2720	[7] Daw, 1973
Jejunal atresia	N/A	2700	[1] Shrand, 1972

*N/A* Not available

Britton and Britton (1995) reported that the mean gastric volume of a normal newborn was 4.9 ± 0.2 mL [12]. In babies with high and low types of intestinal obstruction, the mean gastric aspirate volume was 58.6 ± 6.1 mL [12]. In our patient, the gastric aspirate volume was 35 mL in the delivery room and additional 35 mL was obtained upon admission to the NICU. Although routine determination of gastric aspirate volume is not indicated for all newborns, it may be helpful in the initial evaluation of babies with suspected congenital intestinal obstruction.

There is no reported incidence of BSAF in neonates with congenital intestinal obstruction. Because there are few case reports, it is not common. It is noteworthy that only half of fetuses with esophageal atresia, and two thirds of fetuses with duodenal or proximal jejunal atresia develop polyhydramnios. Questions about AF dynamics remain unanswered [13]. We speculate that bilious regurgitation would occur if there was marked bowel distension secondary to increased fetal swallowing and decreased gastrointestinal (GI) absorption of the AF.

## Conclusions

We report a case of a baby with jejunal atresia who presented with BSAF. A large volume of bilious gastric aspirates was noted in the delivery room and, later, some sticky white meconium was noted in her rectum. Our case is a reminder that the greenish staining of AF could be secondary to *in utero* bilious vomiting or regurgitation and not necessarily due to meconium.
